# Just world beliefs, personal success and beliefs in conspiracy theories

**DOI:** 10.1007/s12144-021-01576-z

**Published:** 2021-03-18

**Authors:** Adrian Furnham

**Affiliations:** Norwegian Business School (BI), Nydalveien, Olso, Norway

**Keywords:** Just world, Conspiracy theories, Success, Happiness, Politics

## Abstract

Do those who believe in conspiracy theories feel less happy and healthy than others? Do they believe the world is simply unjust? This study was concerned with how demographic factors, personal ratings of success, personal ideology (political and religious beliefs) and Just World Beliefs are related to Conspiracy Theories. In total, 406 participants completed two questionnaires: Just World scale (Rubin & Peplau, 1975) and Conspiracy Theories Inventory (Swami et al., 2010) and provided various personal details. The Just World Scale yielded two scores: Just and Unjust beliefs. Participants also reported on their health, happiness and success and a reliable composite measure of well-being was computed. A regression showed younger males, with Unjust World beliefs and politically right-wing views, were more likely to endorse Conspiracy Theories. The discussion revolved around explaining individual differences in accepting these theories. Implications and limitations are discussed.

Those working in the area of Conspiracy Theories (CTs) and Just World Beliefs (JWBs) both argue for the functionality of both belief systems for individuals. They help to make sense of personal and global events and thus give a sense of control and predictability (Stojanov, Bering, & Halberstadt, 2020; Walter & Drochon, 2020). This study examined the relationship between these two belief systems.

## Conspiracy Beliefs (CTs)

CTs are lay theories that attribute the cause or concealment of an event to secret, unlawful, and malevolent processes controlled by multiple actors working together (Zonis & Joseph, 1994). Beliefs in many types of CTs are widespread across the globe (Byford, 2011; Byford & Billig, 2001; Douglas et al., 2019; Douglas, Sutton, & Cichocka, 2017; Swami et al., 2011; van Prooijen, 2020). The area has attracted a great deal of research, covering many topics from classic conspiracies like 9/11, and the disappearance of Amelia Earhart (Swami & Furnham, 2012), to Brexit (Swami, Barron, Weis, & Furnham, 2018) and COVID -19 (Georgiou, Delfabbro, & Balzan, 2020). There are also experimental studies concerning the manipulation of particular CTs (Poon, Chen, & Wong, 2020; Swami, Voracek, Stieger, Tran, & Furnham, 2014).

CTs, like Just World Beliefs, appear to be psychologically functional in that they help individuals attain or maintain a sense of meaning, control and personal security (Newheiser, Farias, & Tausch, 2011). Shermer (2010) argued that CTs are held by people with four particular characteristics: *patternicity* (the tendency to find meaningful patterns in random noise), *agenticity* (the beliefs that the world is controlled by invisible, intentional agent(s)), *confirmation bias* (the strong preference to seek/find conformational evidence for what they believe) and *hindsight bias* (tailoring after-the-fact explanations to what they already know happened).

Early studies traced beliefs in CTs to feelings of powerlessness, particularly among marginalised people, who believe they have become voiceless (Hofstadter, 1965). More recent studies have shown that CTs are associated with political cynicism, authoritarianism and support for democratic principles (Swami, 2012; Swami & Furnham, 2012: Swami et al., 2010). Other early work suggested that beliefs in CTs serve self-esteem maintenance purposes (Robins & Post, 1997), while providing believers an outlet for reasserting their individualism (Melley, 2000) or for the expression of negative feelings (Ungerleider & Wellisch, 1979). Indeed, Van Prooijen, Krouwel, and Pollet (2015) have argued that there is evidence for an adaptive-conspiracism hypothesis, which suggests that CTs are psychological adaptative in that they are functionally designed to deal with specific, recurrent dangers posed by hostile coalitions in human evolutionary history.

Many studies have attempted to examine the individual difference correlates of beliefs in CTs (Abalakina-Paap, Stephan, Craig, & Gregory, 1999; Crocker, Luhtanen, Broadnax, & Blaine, 1999; Goertzel, 1994). For instance, in a pan- European study of over 11,500 individuals Walter and Drochon (2020) found that “people who are male, old, unemployed, positioned at the ideologically extremes, who do not feel represented by parliament, feel economically insecure and use for their news source blogs and nonmainstream social media instead of newspapers have on average a stronger tendency towards conspiracy thinking “(p 15).

There has also been an exponential growth in the research on CTs, as a function of many recent world events and particularly the COVID-19 crisis (Biddlestone, Green, & Douglas, 2020; Georgiou et al., 2020). As a result, there has been theory development as to who, why and when CTs of many sorts are adopted (Swami & Furnham, 2014; van Prooijen, 2020). This has a practical significance as many organisations are interested in combatting CTs, which can have very negative consequences for a society.

Reviews on the origin of conspiracy theories have noted the range of psychological, political and social factors associated with their origin (Douglas et al., 2019; Goreis & Voracek, 2019; Sunstein & Vermeule, 2009; Sutton & Douglas, 2020). CTs have been interpreted from many different theoretical perspectives (Klein, Clutton, & Dunn, 2019) including social representation and frame theory (Franks, Bangerter, & Bauer, 2013). There is an interest in the causes (Oliver & Wood, 2014) correlates (Lahrach & Furnham, 2017) and concomitants of CTs (Freeman & Bentall, 2017).

In a systematic and meta-analytic review, Goreis and Voracek (2019) noted that the psychological studies that examine predictors of CTs are divided into two categories: pathological (i.e. paranoia) or socio-political (perceived powerlessness). This study focusses on the latter approach and may be the first to look at JWBs and CTs.

In general, the earlier psychological studies showed that belief in conspiracist ideas was correlated with anomia, low levels of interpersonal trust, feelings of social and political alienation, and perceptions of being disadvantaged. Recently, Jolley, Douglas, and Sutton (2018) showed that by blaming tragedies, disasters, and social problems on the actions of a malign few, CTs can divert attention from the inherent limitations of social systems. They proposed a *System Justification Theory* which is characterised as an extension of Just World theory, suggesting a relationship between the two. There has also been an interest in the mental health of those who endorse CTs (Furnham & Grover, 2020; Swami, Weis, Lay, Barron, & Furnham, 2016b) which gives further insight into the aetiology of, a “cure” for, or changing of, CTs in the wider society.

## Just World Beliefs

This study is on the belief in a Just/Unjust world (JWB) as predictors of CTs. The JWB concept was identified over 30 years ago and concentrates on the tendency of people to blame victims of misfortunes for their own fate (Lerner, 1980; Lerner & Miller, 1978). The idea is that people have a fundamental need to believe that the (social) world is a just place and that this belief is functionally necessary for them to develop principles of deservingness. There are a number of scales to measure JWBs, including the one used in this study (Rubin & Peplau, 1975), which yields a single score, though it is advisable to calculate a total score for the Just and Unjust World items (Furnham & Gunter, 1984). Two important reviews have appeared (Furnham, 2003; Hafer & Begue, 2005) in this area of research, which attracts 30–50 published papers a year.

People are confronted with difficult social problems such as why some people get ill, are abused, descend into poverty etc. while others do not. They also have to make sense of their own misfortunes. The idea of the JWB is that it helps answer some of these very difficult moral, political and social questions. Many researchers have noted that classic JWB scales have both Just and Unjust items, but that when the two separate scales are combined the negative correlations are modest, and that they are usually quite differently related to other variables. Hence it is advisable to measure each individually. Further it is the Just World, but not Unjust World beliefs, that are adaptive and related to well-being: Unjust World views have the opposite correlates (Bartholomaeus & Stelan, 2019). This is true of beliefs that the world is unjust and that potentially powerful social and political forces control events.

In this study we predict that UJW beliefs will be positively associated with CTs (H1). That is, those who believe the world is Unjust the sense that “bad things happen to good people” are likely to believe in many CTs. The UJW belief reflects the unfairness of the world and a sense of powerlessness which is associated with CT beliefs. There should be no relationship to JW beliefs.

## Personal Characteristics

Studies on CTs have examined many individual difference correlates. The data in this area suggests that more pessimistic people with low self-esteem are more likely to endorse CTs. In this study we asked a number of questions using self-ratings of happiness, health, success at work and wealth. We assumed that these would correlate highly and form a single well-being factor which would be related to CTs. The hypothesis, based on the literature, tested was that there would be a significant negative correlation between self-ratings representing well-being and positive self-regard, and endorsement of CTs (H2). That is, people who feel less successful and adapted search out possible explanations for their condition and CTs can function to do this.

This paper also examined the usual demographic variables of age, sex, education, and also political beliefs. On the basis of many previous studies reviewed, we predicted that males more than females (H3), younger rather than older (H4), and politically right-rather than left-wing people (H5) would have higher CT scores. Many poorly educated young men feel alienated and disenfranchised and are attracted by both right-wing politics and CTs (Swami & Furnham, 2014).

## Method

### Participants

There were 406 American participants, of which 161 were male. They ranged in age from 19 to 60 years (*Mean* = 23.45; *SD* = 12.11). In all, 26% had a high school certificate, 47% an undergraduate degree and 21% a postgraduate qualification. 50% claimed to be Christian and 35% Agnostic or atheist.

### Questionnaires

1. *Belief in Conspiracy Theories Inventory (BCTI):* This is a 15-item scale that measures an individual’s general conspiracist ideation. It was developed by Swami et al. (2010) and the BCTI has a one-dimensional factor structure. Internal consistency coefficients were good in that study (Cronbach’s Alpha = .85) and patterns of correlations with demographic and other belief variables are reported in Swami et al. (Swami et al., 2010; Swami et al., 2011). The Cronbach alpha in this study was .82.
2. *Belief in a Just World*. Rubin and Peplau (1975) devised a self-report inventory to measure the attitudinal continuity between the two opposite poles of total acceptance and rejection of the notion that the world is a just place. The scale has been quoted over 650 times in the academic literature. Although there are newer versions of the scale the original is still often used because of its psychometric qualities (Furnham, 2003). The Cronbach Alpha in this study for the Just World was .88 and .82 for the Unjust World.
3. *Personal information*. In all our studies we ask similar questions in addition to demographics as we have found they are important correlates of important beliefs and behaviours (Furnham, Horne, & Grover, 2020). They are essentially about self-beliefs and ideology and are a parsimonious way of measuring both variables.
  *Self Beliefs*: Participants were asked to rate themselves on a 100-point scale (where 100) is high, they rated their physical attractiveness (*M* = 61.43; *SD* = 19.33); physical health (*M* = 69.06, *SD* = 19.55); current happiness (*M* = 66.97; *SD* = 24.49); wealth (*M* = 42.70; *SD* = 23.32) and success at work (*M* = 57.28; *SD* = 26.42).
  *Ideology:* They rated themselves on a 7-point religious scale (1 = Very to 7 = Not at all) (*M* = 3.23; *SD* = 2.39) and simple one rating politics scale (1 = Very Left Wing to 7 = Very Right Wing) (*M* = 3.57; *SD* = 1.66). This is a simple single judgment which has proved to be theoretically related to other factors (Grover, McClelland, & Furnham, 2020).
  They were asked to rate themselves on a 7-point scale for “I am an optimist” (1 = Strongly Disagree to 7 = Strongly Agree) (*M* = 4.94; 1.57) and “I believe alternative medicine works” (1 = Strongly Disagree to 7 = Strongly Agree) (*M* = 4.39; 1.67).

### Procedure

Ethics permission was sought and received. All data collection was conducted through Amazon Mechanical Turk (MTurk), which has been used in many studies (Buhrmester, Kwang, & Gosling, 2011). Participants were recruited within the framework and guidelines, and had to be American citizens between the ages of 18 and 60 years. Participants were paid £1.00 (around 90c) each for completion. We started with a population of 450 but this was reduced to 406 once the data were cleaned: incomplete responses, evidence of not taking the test seriously, completing the test in an impossibly short time. This is a standard procedure when using data of this sort.

## Results

A number of the personal self-ratings were inter-correlated: See Table 1. Five ratings were positively inter-correlated .29 < *r* < .58 (*N* = 394), which were ratings from 0 to 100 on how happy, happiness, healthy, wealthy, and successful at work they were as well as whether they were an optimist or pessimist. The Cronbach alpha for this variable labelled personal success was .76 which is judged acceptable as a measure of internal reliability. High scores indicate a measure of general self-worth, success and self-esteem.

**Table 1:** Means, SDs and Correlations with between all variables

	Mean	SD	Sex	Age	CT	JW	UJW	Attractiveness	Health	Happiness	Wealth	Work Success	Religion	Politics	
Sex	1.59	.49													
Age	23.45	12.12	.16**												
CT	54.06	28.00	.13**	−.14**											
JW	40.02	7.46	.01	.06	**−.09**										
UJW	37.10	6.20	.06	−.13**	.30**	−.11*									
Attractiveness	61.43	19.33	.02	−.05	**.03**	.12*	−.04		–						
Health	69.10	20.00	−.04	.02	**−.08**	.20**	−.08	.51**							
Happiness	67.00	24.50	.05	.13*	−.13*	.26**	−.18**	.43**	.47**						
Wealth	42.80	23.32	−.12*	−.06	**.00**	.18**	−.02	.43**	.38**	.51**					
Work Success	57.29	26.42	−.11*	.02	−.12*	.21**	−.12*	.35**	.41**	.55**	.58**				
Religion	3.23	2.39	−.19**	−.18**	**−.09**	−.20**	.04	−.03	−.01	−.10	−.10*	−.11*			
Politics	3.57	1.66	−.05	.11*	.12*	.29**	−.04	.06	.05	.09	−.11*	.07	−.39**		–
Optimism	4.94	1.58	.10*	.12*	**.00**	.26**	−.07	.26**	.32**	.55**	.23**	.31**	−.22**	.06	

*p* < .001***, *p* < .01**, *p* < .05*

There were no significant sex differences on any variable, though males scored significantly higher on CT beliefs compared to females (*M* = 57.08 (*SD* = 26.70) vs *M* = 49.60 (*SD* = 29.23); *F*(1, 394) = 6.96, *p* < .01; Effect Size (Cohen’s *d* = .26)). The CT score was correlated with Unjust (*r* = .30, *p* < .01) but not Just World Beliefs. This confirmed H1. It was also correlated with age (*r* = −.14, *p* < 01) (confirming H4), political orientation (*r* = .12, *p* < 01) (confirming H5) and the success variable (*r* = −.12, *p* < .05). The effect sizes for these correlations are however very low.

A regression was then run with CTs as the criterion variable and the above six variables (sex, age, political beliefs, self-rated success/well-being, JW and UJW beliefs) as the predictor variables: See Table 2. This was significant (*F*(6,377) = 11.71, *p* < .001; AdjR^2^ = .14. Four of the six variables were significant: UJW beliefs (beta = .27, *t* = 5.52 *p* < .001); political orientation (beta = .20, *t* = 3.93*p* < .001); sex (beta = .14, *t* = 2.87 *p* < .01) and age (beta = −.13, *t* = 2.73, *p* < .01). This indicated that younger, more right-wing males with UJW beliefs were more likely to endorse CTs. The beta for JW beliefs (beta = −.10, *t* = 1.88, *p* < .06) missed being significant at the accepted p < .05 level. These results thus confirmed H1, H3, H4 and H5.

**Table 2:** Results of Regression onto CT

	Unstandardized B	Beta	t
Sex	7.93	.14	2.87**
Age	−0.31	−.13	−2.73**
Politics	3.29	.20	3.93***
Work Success	−0.07	−.06	−1.26
UJW	1.20	.27	5.52***
JW	−0.36	−.10	−1.88

***p < .001 **p < .01 *p < .05

## Discussion

This study confirmed results in some other studies which have found that sex, age and political beliefs are related to CTs (Swami, 2012; Walter & Drochon, 2020). This study showed that those who said they were on the right-wing of the political spectrum were more likely to endorse CTs, which is in accordance with some studies linking authoritarianism to beliefs in CTs (Swami et al., 2016a, b). However, it is equally conceivable to understand left-wing people also endorsing certain CTs. Indeed, Walter and Drochon (2020) showed it was extremism of the right and left that was associated with endorsement of CTs.

Certainly, CTs have the potential to sow discord, violence and public mistrust, which can divert attention from certain political issues and even undermine democratic debate. It would be interesting to have a more detailed measure of political beliefs, such as an interest in and knowledge about politics, party political preferences and political activism. This may indicate a more nuanced relationship between politics and CTs such that it is extremists beliefs on the left and the right that are associated with a belief in CTs (Furnham et al., 2020). In this sense, endorsement of CTs could viewed as an index of political extremism of both far left and right.

Results showed that although, as predicted, self-rated success was correlated with CTs - that is, the more positive and successful the respondent thought they were, the less they endorsed CTs – this factor ceased to be significant in the regression. This suggests that self-ratings of esteem and success play less of a role in CTs than other factors like ideology and world views. This has been observed by other researchers in the CT area, namely that those who endorse CTs tend to be unhappy, marginalised and less objectively and subjectively successful people. However, it seems from the work of Walter and Drochon (2020) that it is ratings of social factors, rather than self-related, issues that are most related to belief in CTs. That is, an individual’s endorsement of CTs can be seen as a part attempt to justify and bolster their relative lack of social connected and success.

In this study we used an aggregate measure of self-ratings and although we explored individual ratings and their relationship to CTs they did not differ significantly. Clearly this is an area for further exploration: what precise features and factors of self-assessment relate to the belief in CTs and more importantly do these beliefs function at all successfully to bolster self-beliefs? It may be that these self-ratings mediate the relationship between personal alienation and endorsement of CTs.

The study examined whether Just and Unjust world beliefs are related to CTs. Many studies have shown that these beliefs are only weakly negatively correlated and associated with different variables (Furnham, 2003). This study showed that it was only Unjust world beliefs that were associated with CTs. Both UJW and CT beliefs are about wider social-political issues and have in common themes around the powerlessness of individuals to influence events and the general unfairness of many things that occur.

One development in the Just World literature has been to view the JWB as a *healthy* coping mechanism rather than being the manifestation of anti-social beliefs and prejudice (Dalbert, 2001). JWB has been seen as an indicator of mental health which helps explain why people are so eager to maintain their beliefs, which may be their major coping strategy. Belief in a *Just* World is clearly functional for the individual. On the other hand. it is clear that UJW are related to beliefs in CTs which are themselves related to numerous poor coping strategies, just as holding CTs.

This study linked two literatures that have overlapping concepts concerned with people’s views of the world that are seen to be psychological functional. Nearly all of the CT literature has associated conspiracy beliefs with poor coping strategies and a negative world view. Similarly, the JWB literature views those who see the world as unjust tend to be less adapted and happy.

Although most of the hypotheses were confirmed, it was apparent from the correlations that effect sizes were small: in this sense the variables we measured did not explain much of our variance in the major dependent variable: i.e. the belief in CTs. This is indeed a common finding in the literature which has driven researchers to seek out yet more individual difference or sociological variables that correlate more highly and explain more of the variance (Douglas et al., 2017; Sternisko, Cichocka, & Van Bavel, 2020). So far, none have succeeded very well, though all sorts of variables have been considered from personality traits and disorders to ideological beliefs (Douglas et al., 2017) Part of the problem however may lie in the measurement of the CTs. Most measures, like the one used here, simply list a number of well-known theories and ask for endorsement. This may yield quite different findings from studies asking people to list CTs they have heard of, or believe in, or indeed tap into their beliefs about very specific theories (Swami & Furnham, 2012). Equally few if any studies have taken the perspective of those who believe in CTs and examined correlates of those.

What are the theoretical and practical implications of this study? The first is that political and world views are, quite understandably, closely related to CTs which are indeed often themselves political world views, particularly among extremists. Researchers interested in both the origin of, and the best ways to reduce, CTs should concentrate on socio-political attitudes rather than classic personality and other individual difference factors. This involves a more nuanced understanding of an individual’s political beliefs and involvement (Furnham & Fenton-O’Creevy, 2018). The growth of the web will no doubt mean that CTs will grow rather than reduce, as individuals have easy access to new CTs and world views that mirror their own. This is why various bodies have called for a closer policing and monitoring of the web to hopefully reduce the spread of particular ideas and theories.

This study was not without its limitations. We used a convenience sample and all data was self-report which is true of nearly all these studies in the literature (2016a, b; Swami et al., 2010). Also, the BCTI measure of belief is conspiracy theories has few “left-wing” and more “right-wing” theories, which may explain the association between political beliefs and belief in CTs. This suggests it is worth replicating the study with a different measure of CTs. Next, as the regression shows, we were only able to account for 14% of the total variance, indicating that a great deal remains unexplained. Finally, like many other studies in this area it was cross-sectional, which makes it impossible to determine causality: do early JWB beliefs encourage people to accept CTs, or vice versa. Only longitudinal studies, as well as experimental, can answer that question.

## Data Availability

**An** SPSS data file is available from the author upon request.

## Declarations

Participants were volunteers and gave consent. The study was passed by the departmental ethics committee: reference number CEHP/2017/565.
